# Factors Associated with Duration of Overall Treatment Time for Cervical Cancer Treated with Definitive Chemoradiotherapy

**DOI:** 10.7759/cureus.5951

**Published:** 2019-10-21

**Authors:** Vladimir Valakh, Bryan C Coopey

**Affiliations:** 1 Radiation Oncology, Allegheny Health Network Cancer Institute, Pittsburgh, USA

**Keywords:** completion, distance to care, cervical cancer, overall treatment time, chemoradiotherapy, brachytherapy, implant, driving, integrated, high-dose-rate

## Abstract

Introduction

For women with intact cervical cancer treated by definitive chemoradiotherapy, the adverse impact of treatment prolongation is well-established. We aimed to identify potentially modifiable factors associated with the unwanted increase in the time required to complete the prescribed course of therapy.

Methods

We retrospectively examined treatment records of 104 consecutive cervical cancer patients receiving chemoradiation for cervical cancer, which included cervical high-dose-rate intracavitary brachytherapy performed at a single referral center. Association of factors, including distance to care, driving time, and income level with overall treatment time, was explored.

Results

Guideline-concordant treatment duration was achieved in 34% of cases. There was no significant correlation between treatment duration and any of the patient-related characteristics assessed in this study. Transitioning of the radiation oncology physician staffing at the brachytherapy center from a private practice non-integrated group to a large integrated group was associated with a decrease in mean overall treatment time, 61 vs. 68 days, p = 0.007.

Conclusions

We did not identify a modifiable patient-related factor amenable to a financial intervention. Administration of brachytherapy within an integrated system resulted in a shortened duration of the entire course of therapy for intact cervical cancer.

## Introduction

Cervical carcinoma is a major healthcare burden with approximately 13,000 cases diagnosed annually in the United States (US) [1]. Disproportionally, this disease affects underserved populations which lack the ready access to the preventive gynecologic care [2]. For Stages IB2-IVA cervical cancer, the most common treatment entails definitive chemoradiotherapy (CRT): the combination of fractionated external beam radiotherapy (EBRT), concurrent weekly chemotherapy, and high-dose-rate intracavitary brachytherapy [3]. Retrospective data point to the adverse impact of CRT prolongation on cancer control, with the most commonly used cutoff of eight weeks [4-5]. However, the patterns of care studies have consistently revealed that < 50% of women complete CRT within the intended time interval [6-8].

Reasons for CRT prolongation have not been conclusively established. Most research on the subject was carried out before the implementation of concurrent chemotherapy or outpatient brachytherapy. In the modern era, association with increased time to completion of CRT was shown for such disparate factors, such as patient age, development of side effects of CRT, lack of focused patient education about brachytherapy, and delay to perform the first implant [8-10].

A notable feature of definitive CRT for cervical cancer is a large number of therapeutic sessions. A commonly used schedule calls for 25 daily consecutive fractions of EBRT and five bi-weekly implants started towards the end of external beam irradiation and performed separately from EBRT and chemotherapy days. Additional appointments for ancillary testing and procedures, such as repeat diagnostic imaging, EBRT boost, and the temporary insertion of a brachytherapy sleeve, are also needed [11]. The successful delivery of this intense treatment package requires the commitment of substantial financial and temporal resources by the patients and their caregivers.

Our facility has been serving as a cervical implant referral center for a large geographic region which included designated economically distressed areas [12]. We have observed that patients residing in those areas, who also were traveling long distances to receive brachytherapy, were at risk of CRT prolongation through delay in starting and completing implants. We hypothesized that a population existed for which the cost of travel and lodging for brachytherapy constituted a barrier to care. This represented a potential target for a focused financial intervention to improve the quality of healthcare delivery. Therefore, we carried out a single-institutional retrospective study to identify factors associated with the duration of overall treatment time in intact cervical cancer.

## Materials and methods

The study was approved by the Allegheny-Singer Research Institute-West Penn Allegheny Health System (ASRI-WPAHS) Institutional Review Board. One hundred and four consecutive patients who received curative brachytherapy to the intact cervix from 2008 - 2017 at a single hospital-based radiation oncology center were identified. The 2018 Federation of Gynecology and Obstetrics clinical stages ranged from IB1 to IVA [13]. The median age was 55 years (range: 25 - 90).

All patients were treated uniformly, receiving 45 - 50 Gy of EBRT to the pelvis in 25 fractions, concurrent weekly cisplatin chemotherapy, and intracavitary high-dose-rate brachytherapy boost in five sessions, given as an outpatient. EBRT and chemotherapy were administered at our center in 28% of women, while 72% were treated by local oncologists in one of 19 referring centers. For EBRT, either intensity-modulated radiotherapy planning and delivery technique or three-dimensional (3D) conformal radiotherapy was used at the discretion of the radiation oncologists. All brachytherapy was administered at our facility. Insertion of either tandem and ovoids, tandem and ring, or tandem and cylinder (< 10% of cases) apparatuses were facilitated by the temporary placement of a cervical sleeve in the operating room at our hospital [11]. Sleeve insertion surgery was performed either before commencing brachytherapy or on the day of the first implant, per a case-by-case consideration. Fractional brachytherapy doses ranged from 5 to 6 Gy. The delivery of the EBRT boost, when needed (for < 10% of patients), was interdigitated with brachytherapy. The duration of the overall treatment time was calculated as the number of calendar days from the first EBRT fraction to the last implant. Women receiving interstitial brachytherapy were excluded from this study, as were those patients unable to complete five sessions of brachytherapy.

In mid-2011, radiation oncology physician staffing at our center was transitioned from a non-integrated private practice group to a large integrated group [14]. Thus, 56% of patients in this study have received brachytherapy within an integrated delivery care system, while 44% have not.

In our brachytherapy practice, > 90% of patients utilized a private car mode of transportation. Therefore, a key independent variable in this study was the one-way driving distance from the patient’s current residence to the brachytherapy center, calculated using web-based software [15]. Similarly, we recorded expected one-way driving time for each patient, taking into account the typical rush-hour delays for a weekday morning arrival at our center. In addition, the geographical distance to brachytherapy was recorded using the straight-line method [16]. Income level was assessed using the 2015 US Census tract median household income data [17]. The rural versus urban residence distinction using rural-urban continuum codes was not deemed feasible for this study as a large county at the center of our geographic region was composed of the metropolitan and non-metropolitan areas [18]. For this study purpose, brachytherapy during the holiday season, when our center had scheduled days off, was defined as the receipt of at least one implant from the fourth week of November through the first week of January.

Associations between variables were assessed by examining scatter plots and performing Pearson correlation. The means for subgroups were compared using the independent variables T-test. Fisher’s exact test was used to evaluate the distribution of categorical variables. The significance level was set at p = 0.05. A multiple linear regression model using two independent variables was created and reviewed. Statistical analysis was performed using the IBM Statistical Package for Social Sciences (SPSS), version 23, software (IBM Corp., Armonk, NY).

## Results

Patient and treatment characteristics are listed in Table 1. The median duration of CRT was 60 days. Thirty-four percent of patients completed CRT within eight weeks of the treatment start date and 56.7% finished within nine weeks. The average one-way driving time to brachytherapy exceeded one hour. The median household income of the study population was 83% of the national median.

**Table 1:** Patient and Treatment Characteristics EBRT: external beam radiotherapy; N: number; N/A: not applicable; US: United States

Parameter	Value (percentage from the total)	Interquartile range
Median age, years	55	43 - 68
EBRT and brachytherapy at different centers, N	75 (72%)	N/A
Brachytherapy within integrated system, N	58 (56%)	N/A
Median straight line distance to brachytherapy, miles	24	12 - 41
Median driving distance, miles	32	16 - 58
Median driving time, minutes	69	50 - 92
Brachytherapy during the holiday season, N	15 (14%)	N/A
Median household income, US dollars	46,700	41,000 - 56,700
Median treatment duration, days	60	55 - 70

**Figure 1:**
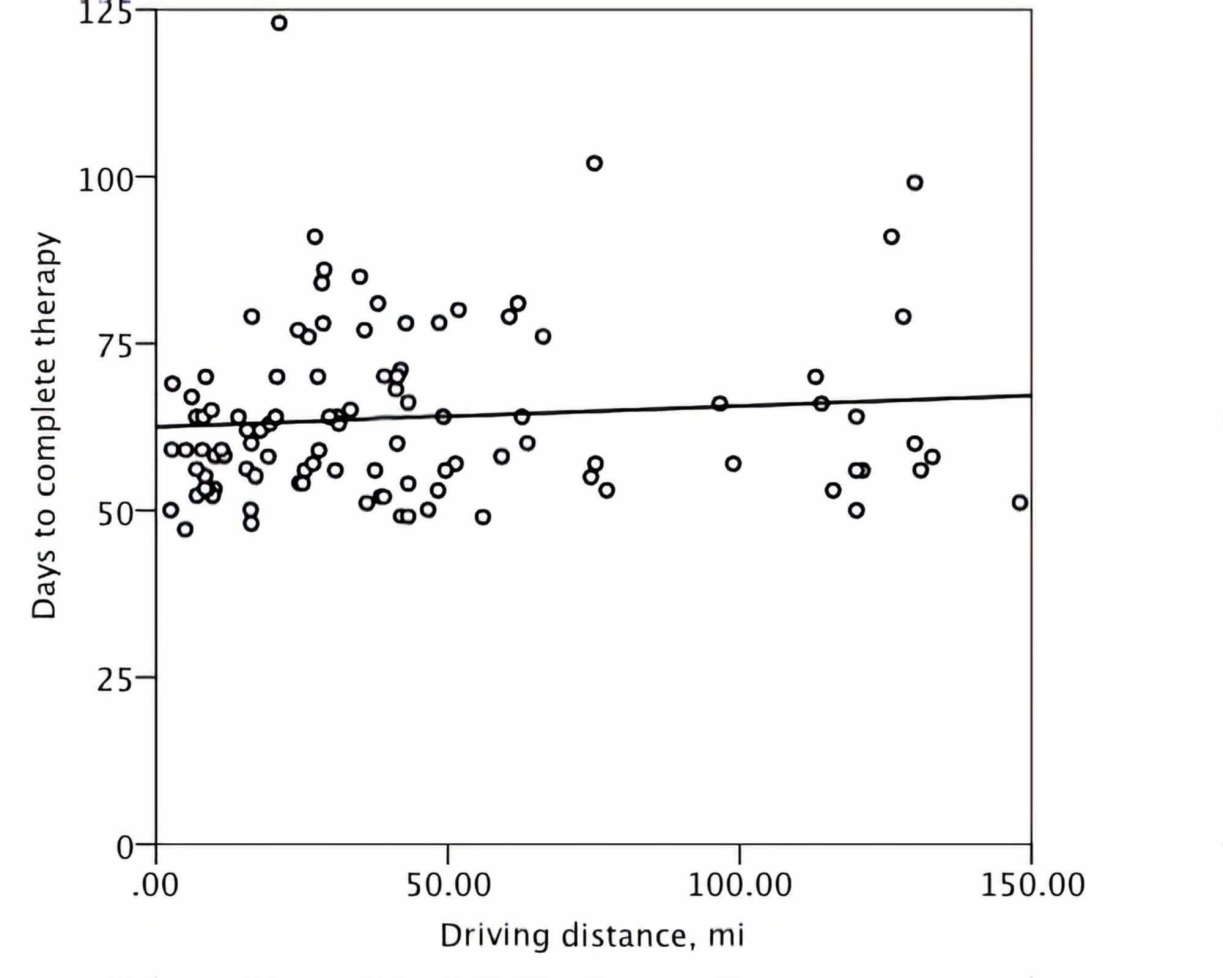
A scatter plot demonstrating the distribution of the treatment duration and one-way driving distance in miles (mi)

**Figure 2:**
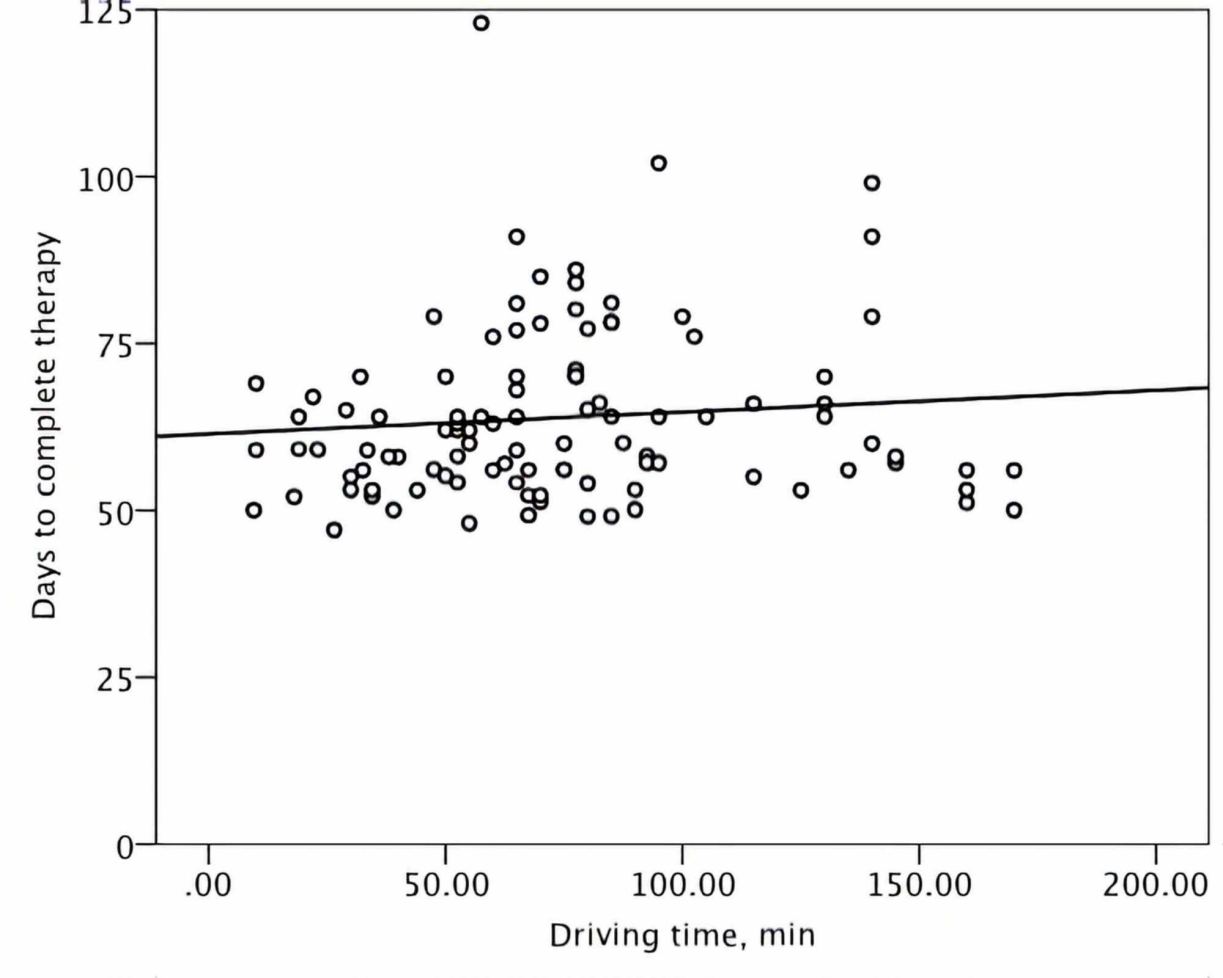
A scatter plot demonstrating the distribution of the treatment duration and one-way driving time to the brachytherapy center in minutes (min)

**Figure 3:**
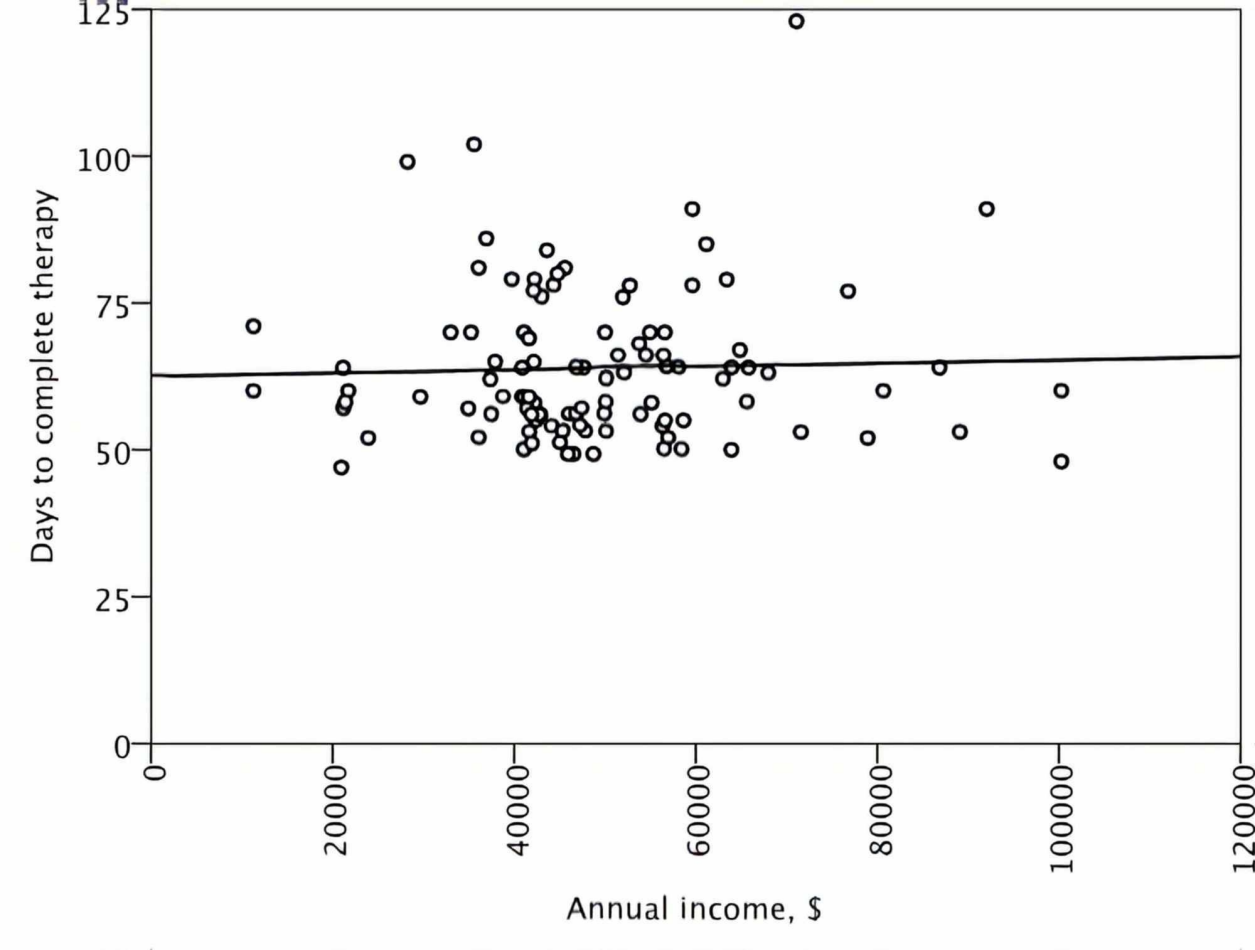
A scatter plot demonstrating the distribution of duration of overall treatment time according to the estimated annual income $: United States dollars

No significant correlation was demonstrated between the treatment duration and either the distance to care, the driving time, or the income level (Figures 1-3). For the patients in the lower quartile for the distance to care, the duration of CRT was statistically significantly shorter in the univariate analysis (Table 2). No association between the level of income and the short distance to care was observed to explain this observation (p = 0.44). Patients living farther away from our center were more likely to receive EBRT and chemotherapy before brachytherapy in the community instead of our center (p < 0.001). However, no influence of the location of the oncology center tasked with EBRT and chemotherapy delivery on the overall treatment duration was present (p = 0.27). Notably, women with the farthest distance to brachytherapy or those in the lower quartile for income were not more likely to experience CRT prolongation compared to the rest of the study population (Table 2).

**Table 2:** Association of Study Variables with Overall Treatment Time EBRT: external beam radiotherapy

Parameter		Mean radiotherapy duration, days	Univariate p-value	Multivariate p-value
Age, years	> 55	65	0.29	
	≤ 55	63		
EBRT at a different center	Yes	65	0.27	
	No	62		
Geographical distance to brachytherapy, miles	> 24	64	0.68	
	≤ 24	63		
Driving distance, miles	> 32	65	0.59	
	≤ 32	63		
Driving distance, miles	> 16	65	0.001	0.49
	≤ 16	59		
Driving time, minutes	> 69	66	0.14	
	≤ 69	62		
Driving time, minutes	> 50	65	0.003	
	≤ 50	59		
Brachytherapy during the holiday season	Yes	63	0.71	
	No	64		
Lower quartile of household income	Yes	66	0.32	
	No	63		
Brachytherapy within an integrated system	Yes	61	0.008	0.007
	No	68		

The administration of brachytherapy within an integrated delivery care system was associated with a shorter duration of overall treatment time (Table 2). Forty-one percent of patients treated in that environment completed CRT within eight weeks vs. 26% who did so before the transition to the integrated system (p = 0.077). In the multivariate analysis, integrated care maintained the association with faster treatment completion while distance did not (Table 2).

## Discussion

This single-institutional retrospective study has demonstrated that the minority of patients with carcinoma of the intact cervix have completed definitive chemoradiotherapy within the timeframe suggested by the current guidelines. Sixty-six percent of women required longer than eight weeks to receive the prescribed therapy, which is similar to the recent large database studies on the modern cervical CRT in the US [7-8]. CRT starts with the first fraction of EBRT and concludes on the day of the last implant. Therefore, prolongation of CRT can occur due to breaks in EBRT, inability to initiate brachytherapy sufficiently early in the treatment program, delays in completion of brachytherapy, or any combination of these three factors.

As most women in this study received EBRT and chemotherapy in a community setting, the precise treatment calendars and the toxicity descriptions could not be collected. However, when those data were available, we have noted that unscheduled EBRT breaks were rare. High-grade treatment side effects after completing EBRT and cisplatin were uncommon as well. It is in line with data demonstrating an excellent tolerance of 45 - 50 Gy of external beam radiotherapy with intravenous cisplatin chemotherapy [19]. Thus, for women with prolonged overall treatment time, non-receipt of timely brachytherapy was the most likely reason.

Based on case observations, we have postulated that patient-related factors were the underlying mechanism of delay in starting and completing brachytherapy. Specifically, we have hypothesized that for a subgroup of patients, the cost of private automobile travel and the overnight lodging for five outpatient brachytherapy sessions constituted a modifiable barrier to care. However, this study demonstrated that overall treatment time was not associated with either driving distance, time, or income level. A similar observation has been reported by Spees et al. [8]. Therefore, a focused financial intervention, such as the creation of an institutional fund to pay for cervical patients’ gasoline and lodging expenses, was not likely to improve the overall treatment time for intact cervical cancer.

Instead, the results of this study suggest physician and program-related factors for non-receipt of timely brachytherapy and, in turn, lengthening of the overall treatment time. Compared to other forms of radiotherapy, cervical brachytherapy has been reported to require a much larger commitment of the physician’s time effort [20]. Further, guideline-concordant brachytherapy for cervical cancer patients who start EBRT and chemotherapy at a different center, as it was done for most of the women in our study, necessitates additional resources for coordinating care between two treating teams. Improved coordination is a feature of integrated healthcare delivery systems [21]. We observed improvement in overall treatment time after transitioning physician staffing to an integrated group.

Lastly, the optimal number of total therapeutic sessions for intact cervical cancer treated with external beam radiotherapy, concurrent chemotherapy, and high-dose brachytherapy may be potentially decreased without affecting treatment efficacy. Supported by the findings of this study, from 2017 onwards, we have aimed to reduce the typical number of intended EBRT fractions and implant insertions from 25 to 22 and from five to four, respectively, while maintaining the same biologically equivalent prescribed radiotherapy dose [22]. That should shorten overall treatment time by one week in an uncomplicated case, potentially improving pelvic cancer control [5].

## Conclusions

The duration of the overall treatment time in cervical cancer treated with definitive CRT constitutes an important measure of the quality of care. This retrospective single-institutional study demonstrated that a minority of patients completed CRT within a time interval suggested by the clinical guidelines. The most likely culprit was the inability to initiate and/or complete cervical brachytherapy in a timely manner. Interestingly, we were unable to identify a patient-related factor for treatment prolongation, which could be addressed through a financial intervention, such as a travel and lodging fund. Instead, performing brachytherapy within an integrated care network was associated with faster completion of CRT for women with cervical cancer. We have concluded that while patients’ cooperation is a prerequisite, improvement in overall treatment time in cervical cancer requires a concerted effort by the physicians and the ancillary healthcare staff.
